# Fiber-Bragg-Grating-Based Displacement Sensors: Review of Recent Advances

**DOI:** 10.3390/ma15165561

**Published:** 2022-08-12

**Authors:** Marco Bonopera

**Affiliations:** National Center for Research on Earthquake Engineering (NCREE), Taipei 10668, Taiwan; marco.bonopera@unife.it

**Keywords:** Bragg wavelength, displacement sensor, fiber Bragg grating (FBG), information detection, monitoring, optical fiber, structure design

## Abstract

With the development of fiber optical technologies, fiber Bragg grating (FBG) sensors are frequently utilized in structural health monitoring due to their considerable advantages, including fast response, electrical passivity, corrosion resistance, multi-point sensing capability and low-cost production, as well as high accuracy and resolution over a long period. These characteristics allow FBG to be a proper alternative sensing element for displacement measurements. In this article, the recent sensing advances and principles of detection of FBG-based displacement sensors are illustrated. Specifically, the latest FBG-based displacement technologies are examined from three principles of detection, i.e., wavelength, intensity and phase signal demodulation. Regarding wavelength detection methods, the problem related to the cross-sensitivity can significantly be reduced depending on the new type of cantilever–FBG-based sensing developed. Vice versa, only the packaging method of FBG prestressed between two fixed ends can still avoid the chirp phenomenon in the reflection spectrum. Moreover, to attenuate the influence of temperature variations on the accuracy of FBG displacement sensors, specific temperature self-compensation structures were successfully designed according to the concepts of phase signal demodulation. In future investigations, different elastic structures and gratings manufactured through special fibers and new methodologies for temperature compensation will still highly refine the efficiency of FBG-based displacement sensors.

## 1. Introduction

The measurement of displacement, strain, vibration and various physical parameters is of essential importance in structural health monitoring (SHM) [1]. With the advancement of fiber optical technologies, fiber Bragg grating (FBG) sensors are frequently utilized in SHM because of their enormous advantages, including fast response, electrical passivity, corrosion resistance, multi-point sensing capability and low-cost production, as well as high accuracy and resolution over a long period. In 1970, a low-loss glass optical fiber was mass-produced by “Corning Incorporated”, an American multinational company. In 1977, the world’s first system based on optical fiber communication was implemented for commercialization in Chicago, United States. Hill et al. [2] discovered the photosensitivity of a germanium-doped optical fiber and adopted an argon-ion laser to cause chemical changes in the photosensitive fiber. Meltz et al. [3] changed the internal molecular of photosensitive fiber with a high-energy ultraviolet laser using a transverse holographic approach, thus making the fiber an optical filter. Subsequently, Hill et al. [4] developed the phase mask method to produce FBG, which led to the maturation of its mass production. Ever since its discovery, FBG has achieved broad attention in the field of optical sensing due to its aforementioned benefits. Nowadays, FBG is consequently a proper alternative sensing element for displacement measurements in several SHM applications, including civil [5,6,7] and mechanical engineering [8], robotics [9] and the aerospace industry [10,11]. Yet, the detection of defects by displacement measurements is crucial for safety determination and health monitoring of a structure, allowing for its decision making in terms of retrofitting and maintenance. Depending on the kind of grating, FBG-based displacement sensors can be long, uniform, chirped, tilted or phase shifted with periodic perturbation of the refractive index inside the core of the optical fiber. Often, to measure vertical or inclined displacements, tilted FBG sensors are also required at different locations of a structure [12]. Specially, they can either be surface assembled or embedded in smart systems. For example, monitoring of a bridge includes multiple FBG sensors positioned over its span girders [13], whilst monitoring of a railway track comprises multiple FBG sensors laid over the track of the railway line [14]. Indeed, one of the high potentials of optical fiber sensing technology is the plain connection of sensors through FBG with distinct reflection wavelengths linked in series and over long distances using a single transmission fiber [15].

In this article, the significant advances in sensing design and principles of detection of FBG-based displacement sensors are presented. Scholars worldwide have carried out investigations to improve their sensing principles, structure design and detection methods along with their deployment in important applications of SHM. Specifically, the recent FBG displacement sensing technologies were examined from three principles of detection, i.e., wavelength, intensity and phase signal demodulation. Regarding wavelength detection methods, the problem related to the strain and temperature cross-sensitivity can significantly be reduced depending on the new type of cantilever–FBG-based sensing developed. Conversely, only the packaging method of FBG prestressed between two fixed ends can still prevent the chirp phenomenon in the reflection spectrum. In addition, to attenuate the influence of temperature variations on the accuracy of FBG displacement sensors, particular temperature self-compensation structures were successfully designed according to the concepts of phase signal demodulation. In Section 2, some general aspects of FBG displacement sensors are introduced. In Section 3, the corresponding advances in wavelength detection methods are classified and described, whilst in Section 4 the advances related to the principles of intensity and phase signal demodulation are discussed. Finally, conclusions and further developments are drawn in Section 5.

## 2. Fiber-Bragg-Grating-Based Displacement Sensors

Displacement is one of the most significant factors because its limit is regularly adopted as a control index of structures [16]. Nevertheless, it is difficult to directly achieve real-time displacement measurements. For instance, an inclinometer is the conventional device utilized for measurement of inclined displacements in the monitoring of slopes. Mostly, displacement using an inclinometer is not measured directly, but it is calculated using a strain-displacement relation that is conjugated with formulas according to the beam theory. Yet, conventional direct techniques such as dial indicators, transducers, levels and total stations are only suitable for short-term monitoring because they require fixed references and, moreover, have limited measurement points [17]. Vice versa, newer whole field instruments, such as accelerometers, GPS and microwave interferometers, were implemented for real-time displacement measurements, but they require installations of extra setup that bring additional costs and measurements or, similarly, they need parameter-displacement conversions combined with calculations [18]. Along this line of thinking, FBGs are a proper alternative for estimating the displacement field of structures and infrastructure. Notably, FBG-based displacement sensors directly estimate the axial strain. Subsequently, to obtain the external displacement, the FBG sensor transforms the axial strain exerted on the FBG into the displacement detecting the changes of wavelength, bandwidth or light intensity and so on. Indeed, the axial strain, exerted on the FBG, gives rise to the variations of the Bragg length while, for the other characteristics, that depends on the amplitude of length and deformations of the FBG for the chirp.

## 3. Advances in Wavelength Detection Methods

**Figure 1:**
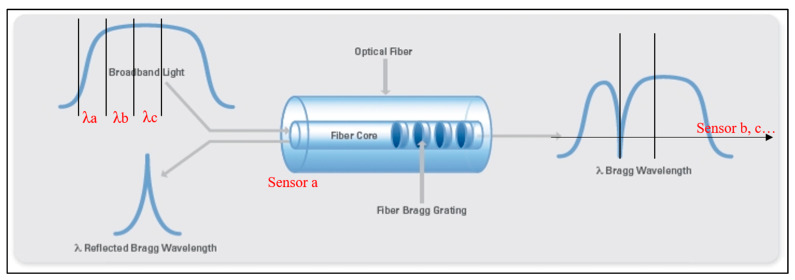
Spectral response for a definite grating period and shifting of Bragg wavelength λ of an FBG subjected to axial loading and temperature. Each of a series of FBG sensors (e.g., sensor a, b, c, …) can occupy a certain broadband light (e.g., λa, λb, λc, …). λ: Bragg wavelength shift.

One type of FBG-based displacement sensor works on the sensing principle of Bragg wavelength shift detection. Based on Bragg condition, variation in Bragg wavelength can be observed, which is representative of the amount of external perturbation (in terms of displacement) exerted on the FBG [19], as depicted in Figure 1.

When a beam of broadband light is incident upon the FBG sensor, the variations in axial strain and temperature result in the Bragg wavelength shifts of gratings, which are utilized as the output signal of the FBG sensor. In fact, the Bragg wavelength of FBG is sensitive to temperature too. To understand how the sensing mechanism of FBG occurs, assume an external constant “*X*”, also called the measurand quantity, which may be the axial strain, temperature or refractive index. The Bragg wavelength of FBG is dependent on this external factor and can be evaluated by differentiating Equation (1) with the measurand constant “*X*” as [20]:

(1) $$\frac{d\lambda }{dX}=2\frac{d\left(\Lambda n_{\mathrm{eff}}\right)}{dX}=2\Lambda \frac{dn_{\mathrm{eff}}}{dX}+2n_{\mathrm{eff}}\frac{d\Lambda }{dX}$$

where *n*_eff_ is the effective refractive index. Since Bragg wavelength is dependent on grating period and *n*_eff_ of FBG, the measurand constant will change either the effective refractive index or grating period of FBG or both, depending on the type measurand. Particularly, the key challenge is the interrogation of the factor signal codified in the wavelength shift. When measurements are conducted in the laboratory, most interrogation approaches use an optical spectrum analyzer (OSA) with a broadband source because it directly executes measurements of the FBG reflection spectrum. The OSA detects the wavelength shift to provide the external displacements, as briefly illustrated in Section 2. However, it is also becoming very common, both in the laboratory and in the prototype phase, to use a simple internal spectrometer, which is a cheaper and compact solution offering a sufficient spectrum discrimination. The advances in wavelength-detecting FBG displacement sensors are classified based on their sensing designs, as will be illustrated in the following sections. Notably, theory, sensing mechanism of FBG and interrogation of FBG sensors were described in detail in Sahota et al. [21].

### 3.1. According to Cantilever Beam Structures

**Figure 2:**
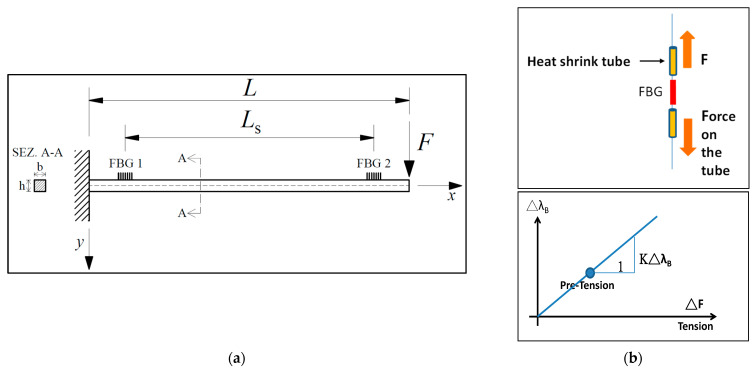
FBG-based displacement sensors according to wavelength shift demodulation: (**a**) Schematic of a proposed cantilever-based sensor. The positions of the two FBGs, at distance *L*_s_ between each other, are shown along with the effective cantilever length *L*; (**b**) mechanism of a proposed two-fixed-ends-based sensor. Heat-shrink tubes at both ends of an FBG that exert an external tensile force F on the FBG (top). Variation in the external tensile force ΔF with variations in the central wavelength Δλ_B_ (bottom).

FBG-based displacement sensors can be composed of various types of transition systems. A cantilever is a conventional elastic element with constant strength, which was theoretically proposed by Guan et al. [22], where the generated strain is equally distributed along its surface. The FBG is indeed fixed on the surface of the cantilever beam structure, as showed in the schematic of Figure 2a. When the free end of the cantilever undergoes an external deformation, the FBG is subjected to a uniform strain. Specifically, the cantilever transforms the displacement of the measured object into a deflection of the beam; consequently, the deflection-induced strain is sensed by the FBG, which brings to a Bragg wavelength variation of the fiber grating. This change in FBG wavelength is linearly proportional to the deflection of the cantilever’s free end. However, in practice, the design uses an equal section cantilever beam rather than a constant strength cantilever beam, which cannot prevent the chirp phenomenon in the reflection spectrum. Thus, the sensitivity of the sensor is slightly lower. Moreover, the strain and temperature cross-sensitivity problem affects the results. Consequently, this kind of system cannot usually reach high precision in displacement measurements. To solve these problems, various technologies to design cantilever–FBG displacement sensors were implemented. In addition, researchers studied the combination of cantilever structures with different elastic elements [23].

Chen et al. [24] linked a hydraulic telescopic cylinder to a cantilever beam in series. Their FBG is formed by a single-mode fiber and a photosensitive fiber. It furnishes two independent Bragg wavelengths for compensation of temperature. The two fibers have distinct responses to distinct external variations. Specifically, the deformations due to the displacements are different, whilst those due to the temperature variation are almost equal. Therefore, the problem related to cross-sensitivity is reduced, and displacements can be measured by the differences of wavelength variations between two FBG wavelengths. This structure made better the limitations associated with the changeability of the elasticity coefficient of force-transmitting media such as springs, and it obtained a measurement range greater than 45 mm with a high sensitivity of 36 pm/mm. This FBG-based displacement sensor can be employed for real-time measurements in several industrial environments such as the mechanical shape or the liquid level monitoring.

To overcome the negative influence of elasticity coefficient changeability of force-transmitting media on the resolution of FBG displacement sensors, Guo et al. [25] studied the combination of a wedge-shaped slider with a cantilever structure. The researchers fixed the cantilever beam’s free end in contact with an inclined plane. When the wedge-shaped slider moves under external deflection, it induces a bending deformation to the cantilever surface. Such a bending strain is sensed by the FBG to estimate the displacement. Meanwhile, the range of measurement and sensitivity of this sensor can be adjusted by modifying inclination and length of the wedge-shaped slider. Its sensitivity is equal to 20.11 pm/mm in the displacement range of 0–100 mm and equal to 123 pm/mm in the range of 0–20 mm. These performances fulfill the requirements of high-precision measurements and long-term stability in the SHM of civil structures and machinery equipment. Furthermore, this FBG sensor was indicated as a promising instrument for micro-displacement measurements, whilst its repeatability and hysteresis errors showed a good creep resistance.

Nazeer et al. [26] proposed a displacement sensor based on an isotropic cantilever structure and according to the principles of interferometry and FBG sensing, as illustrated in the schematic of Figure 2a. Their system adopts a single-mode optical fiber arranged along the cantilever, where two FBGs are located at a certain distance, to estimate the deflected shape of the measured object, making it comparatively an inexpensive approach. On displacing a beam with an unknown magnitude and at an unknown position along its length, Nazeer et al. [26] proved the possibility of measuring the beam-deflected shape and magnitude and location of the external force applied. A calibration approach and an iterative manipulation were used to estimate the two unknowns. Conversely, an analytical solution according to the beam theory was utilized to evaluate the measurement resolution. An accuracy of ±1 mm in a displacement range of 0–20 mm was gained by the researchers.

Li et al. [27] implemented a different kind of pitman FBG displacement sensor having a simple body, small dimension and anti-electromagnetic interference. Specifically, the pitman-style sensor was developed according to a wedge cavity structure which, in turn, was composed of a deflection probe, limit block, spring, pulley, strut, wedge block, metal case, cantilever beam and FBG packaged on its surface. Calibrating data showed that the sensitivity of this sensor is equal to 5.58 pm/mm, and its adjusted R square is up to 0.99, whilst its error is 5.168%. Furthermore, the researchers employed their device in a hysteresis test of a steel frame-reinforced concrete infill wall. It is worth noting that the corresponding measurements matched with those collected from resistance strain displacement meters in a range of 0–50 mm. A part of its good accuracy, this device has a smaller effect on the structure in which it is installed. Its suitability for long term monitoring was also demonstrated.

Instead, Lyu et al. [28] developed a large-range FBG sensor with the goal to measure the bridge beam-gap variations between the beam body of high-speed railway bridges. Traditional sensors are inappropriate to measure this specific gap which, in practice, can reach a value between 200 and 300 mm. This sensor fully exploits the effective space to enlarge the dimension of the mechanical system of the cantilever beam to conduct deflection sensing. Particularly, Lyu et al. [28] simulated such a mechanism using finite element (FE) commercial software. Thus, the sensor was designed, resulting in a simple structure that was easy to package, suitable to extend the range by lengthening the cantilever beam, with good durability, and appropriate for long-term monitoring of large-size beam-gap displacements. When the bridge beam gap changes from 0 to 200 mm, the linear correlation coefficient of the displacement–wavelength curve achieves 0.99852, whilst the deflection estimation sensitivity becomes equal to 4.53 pm/mm.

### 3.2. According to Structures with Two Fixed Ends

Displacements at milli-, micro- or nanoscale are difficult to estimate with high resolutions. This is typical, for instance, in monitoring soil deformation. To overcome this problem, Hong et al. [29] designed a sensor based on an FBG encased in PVC and arranged between two anchorage plates to sense minor displacements causing the two anchorage plates to displace which, in turn, exert on the FBG. To realize the plates and fasten the FBG onto them, fused deposition modeling (FDM) was utilized [21]. Polylactic acid was adopted as a raw material to make anchorage plates with the FDM process. From calibration tests, the range in displacements of this FBG sensor was established between 0 and 0.9 mm, whilst the obtained resolution of minimum deflection was 0.0747 mm. Furthermore, the maximum displacement achieved for horizontal direction was lower than 0.03 mm. This type of FBG displacement sensor can effectively be employed for reporting the flow of vehicles on roads and the kind of vehicle, depending on the wavelength shift. Moreover, its production can support engineers for a better understanding of soil, underlying its utilization in varied applications.

Displacements are difficult to measure in buildings, dams and long-span bridges too. Particularly, vertical deflections of long-span bridges can more easily be recorded by a series of FBG–differential settlement measurement (DSM) sensors linked with a hydrostatic leveling system of communicating containers, as illustrated in Bonopera et al. [30]. This system was designed considering a single acrylate-coated FBG clamped with heat-shrinkable tubes within each vessel, as showed in the schematic of Figure 2a,b. The communicating vessels contain a liquid, and the elastic range of the FBG is controlled by floating mechanics and Hooke’s law. Based on the buoyancy principle, the magnitude of the buoyancy force is equal to the weight of an equal volume of liquid. Thus, as the immersed volume of the suspended mass increases, the force identified by the FBG from pulling the suspended object varies (Figure 2a). Changes in the float buoyancy modify the force exerted in the FBG, therewith changing the Bragg wavelength (Figure 2b). In short, each FBG–DSM sensor provides settlement measurements from the FBG wavelength shift within each vessel. Indeed, the change of the liquid surface (into each container) induces a wavelength variation of the FBG installed. In a range established between 0 and 180 mm, the obtained resolution of minimum displacement was 0.01 mm.

With respect to the above-mentioned developments, Li et al. [31] proposed an FBG sensor with the aim of measuring sub-micrometer displacements for SHM applications. The optical fiber (FBG) is directly prestressed, while its two ends are glued on the sensor frame. A T-shaped cantilever beam and a wedge-shaped slider are also employed by constituting a conversion mechanism. When the measured object undergoes an external deflection, the horizontal displacement is changed into a vertical movement exerted at the FBG’s midpoint. The external deflection is then estimated by the wavelength variation and by the parameters of the conversion mechanism. This sensing structure removed the issues of chirped signal and low repeatability, indicating a sensitivity of 2086.27 pm/mm and a resolution of 0.00048 mm into a range of 1–2 mm. In addition, the chirp phenomenon was removed in the sensor proposed by Xiong et al. [32], where two FBGs are prestressed on two cylindrical rods. A sensitivity of 3304.7 pm/mm and accuracy of 0.02 mm in a displacement range of 0–2 mm were achieved. Therefore, these FBG sensors can significantly be employed for displacement measurements of micro-systems, such as accurate positioning and micro-scaled manufacturing.

The accuracy of displacements of slope profiles utilizing a flexible FBG sensor is mainly affected by its deformations. To effectively mitigate such a limitation, a segmental correction approach according to strain increments clustering was proposed by Tian et al. [33]. A K-means clustering solution was adopted to predict the deformation segments of a flexible FBG sensor with various bending shapes. Consequently, the particle swarm optimization approach was used to estimate the correction parameters corresponding to different deformation segments. FE simulations and tests were executed to validate the advantages of this approach. Experiments showed that the mean absolute errors of reconstructed displacements for six different bending shapes were 1.87%, 5.28%, 6.98%, 7.62%, 4.16% and 8.31%, respectively, which had improved the accuracy by 26.83%, 18.94%, 29.49%, 26.35%, 7.39% and 19.65%, respectively. A resolution in displacement measurements equal to 0.01 mm was obtained.

Another high-precision FBG-based displacement sensor with an embedded spring was developed by Li et al. [34] for SHM of civil structures. Its concept is based on the FBG wavelength variations according to the displacement between measuring points. Calibration tests indicated that the sensor has a good linearity and repeatability. Specifically, a sensitivity coefficient of 23.96 pm/mm and a relative error of 4.94% were obtained after three loading and unloading cycles. Deflection monitoring at very small ranges in quasi-static experiments of civil structures is the most suitable application for this type of FBG sensor.

### 3.3. According to Other Structures

Other sensing structures were implemented with, particularly, the goal of developing new techniques for temperature self-compensating FBG displacement sensors, thus reducing the influence of temperature variations on their measurements. For instance, Thomas et al. [35] presented an FBG sensor for long-range and high-endurance measurement applications in harsh industrial environments. A mechanism was improved which, in turn, is able to convert the deflection of a measurement wire to the elastic displacement of a sensing arm. The difference in wavelength variation between two FBGs symmetrically attached on the sensing arm was discovered by the researchers to be proportional to the displacement. Experiments showed that this structure, respectively, has a sensitivity and a resolution of 23.80 pm/mm and 0.042 mm within a measurement range of 0–150 mm and a good temperature compensation, i.e., with a sensitivity of 0.076 pm/°C within a range of −40–120 °C.

Another temperature self-compensating FBG sensor was implemented by Wu et al. [36] to estimate micro-displacements. Their design is formed by a slider, two flexible springs, a probe and two optical fibers. Such fibers, which are inscribed with FBGs, are tightly suspended in a tilt parallel mode. Particularly, this structure permits the tailoring of the deflection range depending on distinct sensing ranges. Moreover, the discrepancy between the responses of two FBGs is sensitive to the micro axial deformation, whereas it is insensitive to the temperature. Experiments indicated that the displacement sensitivity reaches a value of 1518.6 pm/mm within a range of 0–0.5 mm, whilst a proper capability in micro-displacement monitoring is guaranteed within a temperature variation of 20−50 °C.

Li et al. [37] realized an FBG sensor to estimate vertical deformations of subway floating slabs with the goal to monitor their vibration-reduction and vibration-isolation effects. This sensor is principally made by an optical fiber (FBG), thin-walled ring, steel spring, connecting rod probe, linear bearing, sleeve and supplementary units. The connecting rod probe transfers the external deflection to the steel spring and thin-walled ring and consequently leads to the wavelength variation of the FBG attached in the reserved groove of the thin-walled ring. The linear bearing can decrease losses of friction and simultaneously increase the stability of the sensor. Experiments proved that this FBG sensor has a sensitivity of 36.36 pm/mm and an accuracy of 0.0825 mm into a displacement range of 0−20 mm.

Chen et al. [38] instead presented a new type of dowel bar containing four FBGs for deformation measurements. Their sensing device was principally manufactured for vertical displacement monitoring of joints of concrete pavement slabs because it is prone to arise with time. Relying on the Timoshenko theory, the shear force transferred on the dowel bar and the bending moment on both sides of the joint can be determined based on the deformation of four points along the dowel bar. Therefore, the vertical deflection of the slab joint is estimated by the bending moment and shear force. A curvilinear model between the wavelength drift of FBGs on the dowel bar and the vertical deflection of the slab joint was defined by regression fitting. Furthermore, an analytical–experimental simulation of a full-scale concrete slab into a displacement range of joints of 0−1.0 mm was performed to validate both calculations and the curvilinear model.

Characteristics and load of vehicles that usually cross bridges are crucial to guarantee their structural maintenance and safety. In general, the vehicle load that a bridge is subjected to can be evaluated by the bridge’s structural response in terms of reaction forces at the supports. To assess that, Kim et al. [39] implemented an FBG-based displacement sensor. Particularly, the researchers applied such a device to the Eradi Quake System, i.e., a commercial bridge bearing, to verify its performance and accuracy. In addition to these vehicle loading tests, Kim et al. [39] performed numerical simulations confirming that the vertical displacement from the reaction force at bridge supports is generally a good way to identify vehicle loading.

Notably, in the development of high-precision extensometers, Alias et al. [40] manufactured an embedded FBG sensor using a thermoplastic polyurethane filament within 3D printing technology, taking advantage of its outstanding flexibility and high sensitivity to variations in certain deformations. During evaluation of its performance, the achieved results showed that the sensor can successfully be adopted to estimate displacements with a wavelength responsivity of 1.58 pm/mm and a high linearity (up to 99%). In addition, its integrated protection renders it very suitable for ground displacement monitoring, which can lead to dangerous slippages of sloped earthworks. Furthermore, field testing under underground conditions showed that this FBG-based technology can effectively be applied for measurement of ground movements, offering a wavelength responsivity of 1200 pm/mm under both dry and wet soil conditions, thus providing an optimal configuration of the deflection profile of the earth to be made.

To summarize Section 3, the principal characteristics and information (i.e., method of demodulation, technique, packaging, field application, range of measurement, sensitivity, accuracy and resolution) of the wavelength-detecting FBG displacement sensors, above illustrated, are listed in Table 1. Generally, these sensors are easily multiplexed and do not require complex demodulation schemes, as depicted in Figure 1, but they undergo the relatively lower resolution (usually of ≈1 pm) caused by the use of OSA, as well as by other commercial-off-the-shelf (COTS) interrogators [41]. Compared with the conventional cantilever structures with FBG pasted, the latest advances indicated that, depending on the new cantilever–FBG-based sensing developed, the problem related to strain and temperature cross-sensitivity can considerably be reduced [24], whereas greater ranges of measurement can be obtained [24,28]. Conversely, only the packaging method of FBG prestressed between two fixed ends can still prevent the chirp phenomenon in the reflection spectrum [31,32]. Instead, to decrease the influence of temperature variations on the accuracy of FBG displacement sensors, different structures for temperature self-compensation were designed, ensuring a good stability for long-term monitoring [35] and also reaching measurements of micro-displacements [36]. In addition, the advances showed the approach of using FE simulations to validate the performances of the proposed mechanisms [28,33]. Yet, some of the wavelength-detecting FBG sensors, which were recently implemented, satisfy the requirements of high precision for SHM applications, especially for micro- and sub-micrometer displacement measurements [25,31].

## 4. Advances in Other Detecting Methods

FBG-based displacement sensors were also fabricated according to other detecting concepts and principles. In this article, the advances of two types of detecting methods were treated, i.e., those related to the principles of optical intensity and phase signal demodulation.

### 4.1. Intensity Detecting Methods

The fundamental concept of an intensity signal demodulation FBG sensor is to transform the FBG wavelength variation into an intensity variation, which can be used to define the wavelength location of the sensor [42]. Due to their advantages including simplicity and potential low cost, intensity modulated FBG sensors have attracted significant interest. Compared to the wavelength modulated ones (Section 3), a larger number of parameters can be estimated with the use of lower expensive light sources, simple detection schemes and interrogators while still benefiting from the intrinsic advantages of photonic sensors.

Zou et al. [43], according to the edge filter demodulation method, proved the applicability of a wide range FBG displacement sensors according to an asymmetric twin-core fiber. The edge filter is composed of the fusion splicing of a segment of the twin-core fiber between two single-mode fibers that, in turn, transforms the wavelength variation into an intensity variation. Experiments indicated that the variation in external deflection linearly varies with the output intensity of the sensor. Thus, the deflection can be estimated by measuring the intensity. This sensor uses an edge filter, which gives an output intensity closely related to the Bragg wavelength of input signal. A quick response can be reached.

Likewise, according to the concept of intensity detection, Ghaffar et al. [44] developed an FBG displacement sensor with a simple structure that is easy to manufacture and low-cost. Particularly, the proposed technology relies on the intensity variation between a first and a second optical fiber. Indeed, the sensing system holds a plastic optical fiber with a large and a small diameter. A hole is constructed inside the core of the optical fiber with a larger diameter, and the optical fiber with a smaller diameter is positioned into the hole for displacement measurements. Yet, a tapered fiber is utilized to intensify the coupling made with the heat and drawing process. Experiments showed that the range of this sensor relies on the length of the hole, whereas sensitivity decreases as the length of the hole increases. A resolution of 5.058 × 10^−5^ mm was obtained within a measurement range of 0–1.3 mm, whilst a resolution of 8.16 × 10^−6^ mm was achieved within 1.6–2 mm.

### 4.2. Phase Detecting Methods

The unbalanced Mach–Zehnder interferometer (MZI) demodulation method is a signal conversion approach that transforms the FBG wavelength shift into phase variation, which can be adopted for detection [45]. According to this process, an FBG displacement sensor, which uses a slow light interferometer, was improved by Zhang et al. [46]. The optical fiber MZI, based on slow light in a polymer-infiltrated photonic crystal waveguide (PI-PCW), was introduced to improve the sensitivity of demodulation of the FBG sensor. Optimizing the structure of the PI-PCW, a slow light with a high group index of 110 was executed. Due to the electro-optic effect of the infiltrated polymer, the working wavelength of flat band slow light, as well as the demodulation range of the MZI, can be flexibly modified and enlarged by tuning the external driving voltage. Lastly, a differential and orthogonal approach was utilized to demodulate the interference spectrum of the MZI with a higher stability and proper linearity. The FBG was attached on an Omega-like beam structure, so the deflection variation of free end of the Omega-like beam can be evaluated by monitoring the output phase of the MZI. In addition, this sensor showed a good linearity, a high sensitivity of 1.035 rad/mm and a wide range of displacement measurement equal to 55.6 mm.

The other three types of FBG sensing systems based on the principle of phase signal demodulation are worth being illustrated. According to the Fabry–Pérot (FP) effect of FBG, a temperature-insensitive FBG displacement sensor and a peak wavelength demodulation approach were introduced by Tao et al. [47]. Owing to its advantages of compactness and temperature independence, this device has a sensitivity of 117 pm/mm and an accuracy of 0.085 mm into a range of 0–2 mm. An apodized FBG was glued at a certain location on the inner surface of a thin-walled ring. When the ring is under deformation, the FBG is split into two segments of equal FBGs but oppositely directed chirp gradients. From this, the FP cavity into the grating area can be constructed, and the resonant peaks can be noticed in the FBG reflection spectrum. The wavelength separation between the wavelengths of the resonant peaks linearly varies with the deflection variation, whilst this separation is insensitive to the change of temperature. In addition, the ambient temperature can be achieved through a simple manipulation, resulting in a sensitivity of 28.67 pm °C^−1^. Nevertheless, another way for acquiring displacements with a high resolution is using the wavelength scanning laser with an FBG Fabry–Pérot interferometer (FPI), as proposed by Zhang et al. [48]. This approach works on scanning of radio frequency signals. The methodology measures displacements at a micro-scale level and furnishes a resolution in nanometers utilizing two FBGs, which in turn forms an FPI cavity.

The last temperature-independent FBG displacement sensor, which utilizes a magnetic scale as a transferring mechanism, was designed by Zhu et al. [49]. Specifically, two FBG wavelength shifts need to be detected, and a full range measurement is gained. For identification of the clockwise and counterclockwise rotation directions of the motor, there is a sinusoidal relationship between deflection and wavelength shift. As the anticlockwise rotation alternates, the wavelength variation of the second FBG detector shows a leading phase difference of around 90° (+90°). As the clockwise rotation alternates, the wavelength shift of the second FBG exhibits a lagging phase difference of around 90° (−90°). Simultaneously, the two FBG sensors are temperature-independent. When a remote monitor without any electromagnetic interference is required, this technology makes it a useful device for deflection estimations. Even if the structure of its sensing structure is quite simple, this FBG sensor is particularly suitable for long-distance measurements in both industrial production and basic research.

To summarize Section 4, the principal characteristics and information (i.e., method of demodulation, technique, packaging, field application, range of measurement, sensitivity, accuracy and resolution) of the FBG displacement sensors according to the concepts of optical intensity and phase signal demodulation are listed in Table 2. In short, recent advances have allowed these sensors to have high precision and good linearity for SHM applications and also the capacity of providing measurements at a micro-scale level [48]. Furthermore, two specific phase-signal-detecting FBG sensors were designed to guarantee their temperature-sensing self-compensation during monitoring [47,49].

## 5. Conclusions

In this article, the important advances in sensing design and principles of detection of FBG-based displacement sensors were analyzed. FBG has rapidly moved from being for the sake of laboratory research to implementations in optical sensing structures after its development. Scholars worldwide have carried out investigations to improve their sensing principles, structure design and detection methods along with their deployment in important applications of SHM. Specifically, the recent FBG displacement sensing technologies were examined from three principles of detection, i.e., wavelength, intensity and phase signal demodulation. Regarding wavelength detection methods, the problem related to the strain and temperature cross-sensitivity can significantly be reduced depending on the new type of cantilever–FBG-based sensing developed. Conversely, only the packaging method of FBG prestressed between two fixed ends can still prevent the chirp phenomenon in the reflection spectrum. In addition, to attenuate the influence of temperature variations on the accuracy of displacement measurements, particular temperature self-compensation structures were successfully designed according to the concepts of phase signal demodulation. In conducting future research, the real-time problems of temperature compensation should still be taken into consideration because several field situations can present an ambient temperature that varies frequently. Yet, there is currently a trend about the miniaturization of multiple configurations of FBG displacement sensors, which focus on novel elastic structures, such as all-fiber systems. In conclusion, with the quick implementation of optical fiber manufacturing technology, special fibers (photonic crystal fibers, micro- and nanofibers, etc.) with a smaller diameter, higher tensile strength and better characteristics are being developed with the aim to still refine the efficiency of FBG-based displacement sensors.

## Figures and Tables

**Table 1:** Applications and performances for FBG-based displacement sensors according to wavelength shift demodulation (Section 3).

Reference	Method of Wavelength Demodulation	Technique	Packaging	Application	Range (mm)	Sensitivity (pm/mm)	Resolution (mm)	Accuracy (mm)
Chen et al. [24]	Cantilever beam structure	Hydraulic telescopic cylinder linked to a cantilever	FBG formed by a single-mode fiber and photosensitive fiber	Displacements in industrial environments	0~45	36.00	—	—
Guo et al. [25]	Cantilever beam structure	Slider combined with a cantilever	Bending strain of cantilever sensed by FBG	Micro-displacements monitoring	0~100 0~20	20.11 123.00	—	—
Nazeer et al. [26]	Cantilever beam structure	Interferometry and FBG sensing	FBG sensing along the cantilever	Any beam of any material	0~20	—	—	±1
Li et al. [27]	Cantilever beam structure	Wedge cavity sensing structure	—	Displacements of civil structures	0~50	5.58	—	—
Lyu et al. [28]	Cantilever beam structure	—	—	Displacements of high-speed railway bridges	0~200	4.53	—	—
Hong et al. [29]	Structure with two fixed ends	Anchorage plate and PVC tube	FBG sealed with PVC tube	Soil strain monitoring	0~0.9	—	0.0747	—
Bonopera et al. [30]	Structure with two fixed ends	Hydrostatic system of communicating vessels	FBG encapsulated in vessels	Long-span bridge displacements	0~180	—	0.01	—
Li et al. [31]	Structure with two fixed ends	T-shaped cantilever and slider	Prestressed FBG bonded from ends	Sub-micrometer displacements of micro-systems	1~2	2086.27	0.00048	—
Xiong et al. [32]	Structure with two fixed ends	Two FBGs prestressed on two cylindrical rods	Prestressed FBG bonded from ends	Crack monitoring	0~2	3304.70	3.03 × 10^−5^	0.02
Tian et al. [33]	Structure with two fixed ends	Flexible FBG sensor	Bending deformation of flexible FBG	Displacements of slope profiles	—	—	0.01	—
Li et al. [34]	Structure with two fixed ends	FBG with embedded spring	FBG wavelength shifts	High-precision displacements of civil structures	—	23.96	—	—
Thomas et al. [35]	Other structure	Wire combined with a sensing arm	Two FBGs attached on sensing arm	Displacements in industrial environment	0~150	23.80	0.042	—
Wu et al. [36]	Other structure	Two FBGs combined with mechanical units	Two FBGs suspended in a tilt parallel mode	Displacements at micro-scale	0~0.5	1518.60	—	—
Li et al. [37]	Other structure	FBG combined with mechanical units	FBG attached on thin-walled ring	Displacements of subway floating slabs	0~20	36.36	—	0.0825
Chen et al. [38]	Other structure	Dowel bar containing four FBGs	FBG strains of four points on dowel bar	Displacements of pavement slabs	0~1	—	—	—
Kim et al. [39]	Other structure	—	—	Detection of load of bridge vehicles	—	—	—	—
Alias et al. [40]	Other structure	Embedded FBG	Wavelength shifts of embedded FBG	High-precision monitoring of ground movements	—	1.58	—	—

**Table 2:** Applications and performances for FBG-based displacement sensors according to optical intensity and phase signal demodulation (Section 4).

Reference	Method of Signal Demodulation	Technique	Packaging	Application	Range (mm)	Sensitivity	Resolution (mm)	Accuracy (mm)
Zou et al. [43]	Intensity method	Twin-core optical fiber between two single-mode optical fibers	Intensity variation between two single-mode optical fibers	High-precision displacement monitoring	—	—	—	—
Ghaffar et al. [44]	Intensity method	Plastic optical fiber with a large and a small diameter	Intensity variation of plastic optical fiber	High-precision displacement monitoring	0~1.3 1.6~2	1.977 nW/μm 12.25 nW/μm	5.058 × 10^−5^ 8.16 × 10^−6^	—
Zhang et al. [46]	Phase method	Optical fiber MZI based on slow light in PI-PCW	FBG attached on Omega-like beam	High-precision displacement monitoring	0~55.6	1.035 rad/mm	—	—
Tao et al. [47]	Phase method	Fabry–Pérot (FP) effect of FBG	Apodized FBG glued on a thin-walled ring	High-precision displacement monitoring	0~2	117 pm/mm	—	0.085
Zhang et al. [48]	Phase method	Wavelength scanning laser with FBG FPI	Scanning of radio frequency signal using two FBGs	Monitoring of micro-displacement with ultrahigh resolution	—	35.70 MHz/μm	—	—
Zhu et al. [49]	Phase method	Magnetic scale, as transferring mechanism, combined with two FBGs	Phase variation between two FBGs	Displacement monitoring in research and industry	—	—	—	—

## Data Availability

Some or all data or information of the manuscript are available from the corresponding author by request.
